# Role of *Klf4* in the Regulation of Apoptosis and Cell Cycle in Rat Granulosa Cells during the Periovulatory Period

**DOI:** 10.3390/ijms20010087

**Published:** 2018-12-26

**Authors:** Hyeonhae Choi, Jaesook Roh

**Affiliations:** Laboratory of Reproductive Endocrinology, Department of Anatomy and Cell Biology, College of Medicine, Hanyang University, Seoul 133-791, Korea; dalpu2002@hanyang.ac.kr

**Keywords:** *Klf4*, granulosa cells, LH surge, apoptosis, cell cycle

## Abstract

In the ovary, the luteinizing hormone (LH) surge suppresses the proliferation and induces the luteinization of preovulatory granulosa cells (GCs), which is crucial for the survival of terminally-differentiated GCs. *Krüppel-like factor 4* (*Klf4*) has been shown to play a role in regulating the cell cycle and apoptosis in various cell types. The rapid induction of *Klf4* expressions by LH was observed in preovulatory GCs. To evaluate whether *Klf4* affects GC proliferation and survival, primary rat GCs were isolated from pregnant mare serum gonadotropin-primed Sprague–Dawley rat ovaries and transfected with a *Klf4* expression vector or *Klf4*-specific siRNA, followed by determination of the transcript levels of apoptosis-related and cell cycle-related genes. Cell proliferation, viability, and apoptosis were analyzed by BrdU incorporation, a Cell Counting Kit-8 assay, a bioluminescence caspase 3/7 assay, and flow cytometry. LH treatment increased *Klf4* mRNA expression in preovulatory GCs. Transcripts of *B-cell lymphoma 2* (*Bcl-2*) and cell cycle promoters (*Cyclin D1* and *Cyclin D2*) decreased, whereas those of the cell cycle inhibitor, *p21*, increased. Altering the expression of *Klf4* by overexpression or knockdown consistently affected the expression of *Bcl-2* and *Cyclin D1*. In agreement with this, *Klf4* overexpression reduced cell viability, increased the fraction of apoptotic cells, and arrested cell cycle progression in G1 phase. We conclude that *Klf4* increases the susceptibility of preovulatory GCs to apoptosis by down-regulating *Bcl-2*, and promotes LH-induced cell cycle exit. It appears to be a key regulator induced by the LH surge that determines the fate of GCs in preovulatory follicles during the luteal transition.

## 1. Introduction

Whether the follicles are growing or atretic depends on the balance between anti-apoptotic and pro-apoptotic factors that determine the rate of granulosa cell (GC) apoptosis. Although apoptosis can occur at any stage of follicular development, follicles that are more differentiated are more prone to apoptosis than early-stage follicles [1]. In addition, the factors regulating follicular cell survival before and after the luteinizing hormone (LH) surge appear to differ. In response to the LH surge, the expression of diverse genes is rapidly and dramatically changed in preovulatory follicles [2]. For instance, highly expressed genes in growing preovulatory follicles are turned off by the LH surge, whereas various different genes that are relevant to luteinization increase dramatically during the periovulatory period [3].

The *Krüppel-like factor* family of transcription factors plays important roles in many biological processes, including proliferation, apoptosis, and differentiation [4,5]. *Krüppel-like factor 4* (*Klf4*) expression was first reported in the epithelial lining of the gut and skin, and its effect on cell cycle arrest and apoptosis is different depending on the cellular context [6,7,8]. An LH-induced up-regulation of *Klf4* expression in porcine GCs in vitro has been reported [9], and it is involved in apoptosis in several mammalian cell types [10]. Since luteinizing GCs cease to multiply, it is conceivable that *Klf4* is induced by LH and influences the cell cycle transition and apoptosis of periovulatory GCs during this critical period. However, there is no direct evidence for anti-apoptotic or pro-apoptotic actions of *Klf4*, or its effects on cell cycle arrest, and its role in the ovary is largely unknown.

In this study, we investigated whether *Klf4* is regulated by LH and whether it is involved in cell cycle arrest and the loss of cell viability during the luteal transition period in rat preovulatory GCs in vitro. Cell cycle arrest was analyzed by measuring the induction of cell cycle-related genes such as *Cyclin D1*, *D2*, *p21*, and *p27* along with an analysis of 5-bromo-2′deoxyuridine (BrdU) incorporation and flow cytometry. Cell viability was analyzed by examining the expression of apoptosis-regulating genes, B-cell lymphoma 2 (*Bcl-2*) and *Bcl-2-associated X* (*Bax*) [11], accompanied by a cell counting kit-8 assay (CCK-8), a sensitive bioluminescence caspase 3/7 assay, and flow cytometry analysis. Here, we found that *Klf4* increases the susceptibility of preovulatory GCs to apoptosis by down-regulating *Bcl-2*, and promotes LH-induced cell cycle exit.

## 2. Results

### 2.1. LH Regulates the Expression of Klf4, Bcl-2, and Cell Cycle Genes in Preovulatory GCs

**Figure 1:**
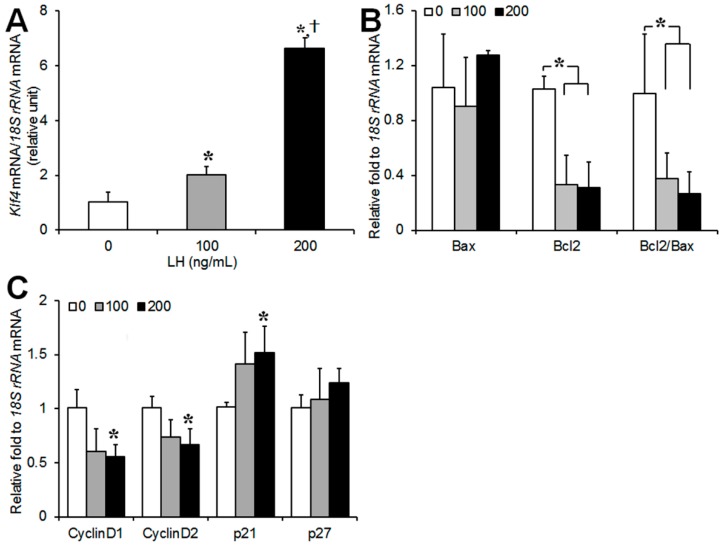
Effect of luteinizing hormone (LH) on *Krüppel-like factor 4* (*Klf4*), transcripts of apoptosis- and cell cycle-related genes in cultured preovulatory granulosa cells (GCs). Real-time RT-PCR analysis of transcripts of (**A**) *Klf4*; (**B**) *Bax*, and *Bcl-2*; and (**C**) *Cyclins D1*, *D2*, *p21*, and *p27* in preovulatory GCs in response to LH (0 ng/mL, 100 ng/mL, and 200 ng/mL). All of the expression levels were normalized to *18S rRNA* levels. Values were calculated as fold changes relative to untreated cells and are expressed as means ± standard deviations (SDs) of three separate experiments. LH, luteinizing hormone. * *p* < 0.05, vs. untreated cells; ^†^ *p* < 0.05, vs. cells treated with 100 ng/mL of LH.

To investigate changes in the expression of *Klf4* and the genes regulating apoptosis and the cell cycle in preovulatory GCs in the early phase of LH exposure, cells were cultured in the presence of LH (0, 100, 200 ng/mL) for 45 min, and real-time PCR analysis was performed (Figure 1). The culture time of 45 min was based on preliminary measurements of *Klf4* expression after LH exposure for 15 min to 24 h. As shown in Figure 1A, LH treatment led to dose-dependent increases in *Klf4* mRNA. For example an ovulatory dose of LH of 200 ng/mL led to a 6.6-fold increase in the *Klf4* mRNA level compared to untreated control cells. LH treatment reduced the expression of *Bcl-2* and the *Bcl-2*/*Bax* ratio in a dose-dependent manner, whereas no clear changes were found in the *Bax* transcript levels compared to the control (Figure 1B). LH treatment decreased the expression of cell cycle promoters (cyclin D1 and D2), and increased that of p21 at 200 ng/mL (Figure 1C). 

### 2.2. Effect of Klf4 on Expression of Apoptosis-Related and Cell Cycle-Related Genes in Preovulatory GCs

**Figure 2:**
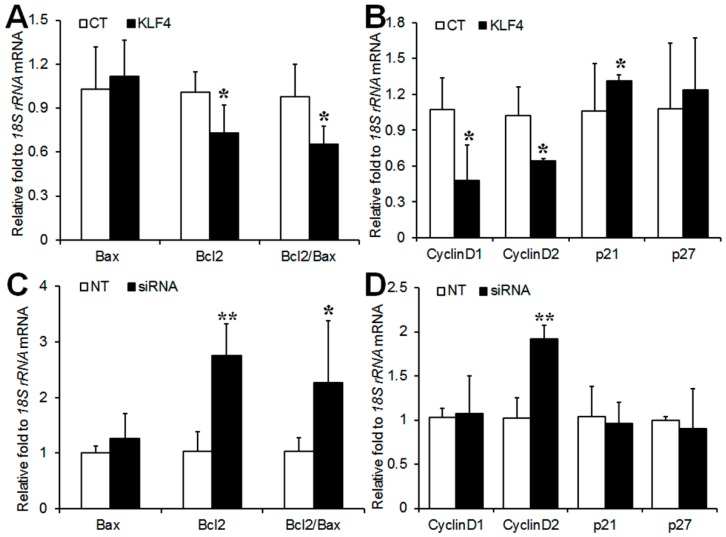
Regulation of apoptosis-related and cell cycle-related genes expression by *Klf4* in preovulatory GCs. (**A**,**C**) Real-time RT-PCR analysis of *Bax* and *Bcl-2* mRNA levels in GCs transfected with *Klf4* expression vector (300 ng) and *Klf4*-specific siRNA (200 nM); (**B**,**D**) Real-time RT-PCR analysis of *cyclins D1*, *D2*, *p21* and *p27* mRNA levels in GCs transfected with *Klf4* expression vector and *Klf4*-specific siRNA. *18S rRNA* was used to normalize each reaction. Values are calculated as fold changes relative to empty vector (CT) or non-target siRNA (NT), and are expressed as the means ± SDs of three separate experiments. CT, cells transfected with empty vector; NT, cells transfected with non-target siRNA. * *p* < 0.05, ** *p* < 0.01 vs. CT or NT.

GCs from pregnant mare serum gonadotropin (PMSG)-primed rat ovaries were transfected with a *Klf4* expression vector or *Klf4*-specific small interfering RNA (siRNA). The overexpression or knockdown of *Klf4* in GCs was confirmed by real-time reverse transcription polymerase chain reaction (RT-PCR) and Western blotting analysis (Supplementary Figure S1). *Klf4* overexpression caused significant decreases in *Bcl-2* transcripts, which were accompanied by reduced *Bcl-2*/*Bax* ratios, with no effect on *Bax* expression itself (Figure 2A). Conversely, *Klf4* knockdown increased *Bcl-2* mRNA expression and the *Bcl-2*/*Bax* ratio (Figure 2C). *Klf4* overexpression down-regulated *cyclins D1* and *D2*, and up-regulated the cell cycle inhibitor *p21* (Figure 2B), whereas only *cyclin D2* was significantly up-regulated in response to *Klf4* inhibition (Figure 2D), suggesting that *Klf4* blocked cell cycle progression primarily by down-regulating *cyclin D2*. 

### 2.3. Klf4 Overexpression Reduces Cell Viability and Proliferation of GCs

**Figure 3:**
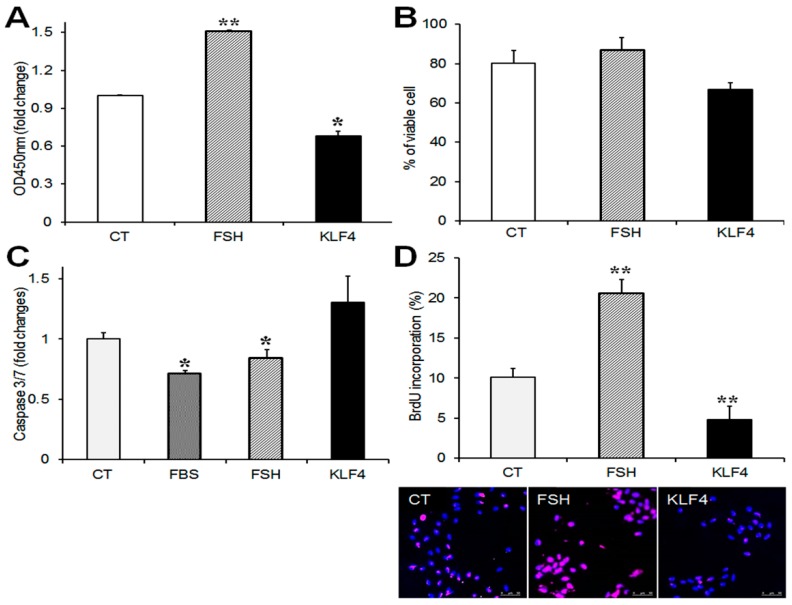
Effects of *Klf4* overexpression on cell viability and proliferation of preovulatory GCs. Cell viability was determined by (**A**) CCK-8 cell counting assay (ALX-850-039-0100, Enzo) and (**B**) trypan blue exclusion in cells transfected with *Klf4* expression vector (300 ng/well) or empty vector (CT, control). * *p* < 0.05, ** *p* < 0.0001 vs. CT; (**C**) Analysis of *caspase 3/7 activity*. GCs transfected with *Klf4* or empty vector were incubated without serum, and some control cells were treated with fetal bovine serum (FBS) or follicle-stimulating hormone (FSH) as positive controls for apoptosis suppressors. Twenty-four h later, *caspase 3/7* activity was measured with the bioluminescence assay. FBS, 10% fetal bovine serum. * *p* < 0.05 vs. CT; (**D**) 5-bromo-2′deoxyuridine (BrdU)-incorporation assay. DNA labeling indices were determined by the scoring number of BrdU(+) nuclei per total nuclei, and are presented as means ± SDs. ** *p* < 0.001 vs. CT. Representative fluorescence microscopy images of BrdU-labeled GCs (100×) (lower panel). Cells were stained with BrdU antibody (B2531, Sigma, 1:200) to visualize BrdU-labeled cells (pink) and counterstained with 4′,6-diamidino-2-phenylindole (DAPI) to confirm nuclear status (blue). CT, control cells transfected with empty vector; FSH, follicle-stimulating hormone (50 ng/mL); KLF4, cells transfected with *Klf4* (300 ng).

To determine the effects of *Klf4* on GC viability and proliferation, cells were transfected with Flag-*Klf4* or empty vector (CT) and cultured for 24 h without serum to avoid any effects on the growth factors in serum. FSH was used as a positive control [12]. The overexpression of *Klf4* in the GCs was confirmed by real-time PCR (Supplementary Figure S1C). The CCK-8 assay was used to monitor the effect of *Klf4* on GC viability. *Klf4* overexpression decreased CCK-8 activity by 30% compared to the control (*p* = 0.035 vs. CT) (Figure 3A). Since the CCK-8 assay measures the metabolic activity of living cells and does not assess cell death, cell viability was measured by trypan blue exclusion. The percentage of viable cells fell by approximately 14% in *Klf4*-overexpressing cells (from 80.3 ± 6.4% to 66 ± 3.3%) (Figure 3B). We also analyzed apoptosis quantitatively by measuring *caspase* 3/7 activity. Transfected GCs were cultured with or without serum or follicle-stimulating hormone (FSH). The enzymatic activities that were measured in each group were compared with the activities in the control cells. As shown in Figure 3C, in the GCs cultured with 10% fetal bovine serum (FBS) and FSH, *caspase* 3/7 activity was 0.7-fold and 0.8-fold of the activity in the control, respectively (*p* < 0.05). The overexpression of *Klf4* caused an approximately 1.3-fold increase in *caspase*-3/7 activity compared to the control cells (*p* = 0.05). 

After FSH treatment, BrdU-labeled GCs increased twofold relative to non-treated control cells (*p* < 0.01), whereas they declined to half the control level of 10.1 ± 1.1% among the *Klf4*-overexpressing cells (4.8 ± 1.7%) (*p* < 0.01) (Figure 3D). These results indicate that *Klf4* is implicated in the regulation of cell survival and proliferation in preovulatory GCs. 

### 2.4. Klf4 Overexpression Induces Apoptosis and Promotes Cell Cycle Arrest in GCs

**Figure 4:**
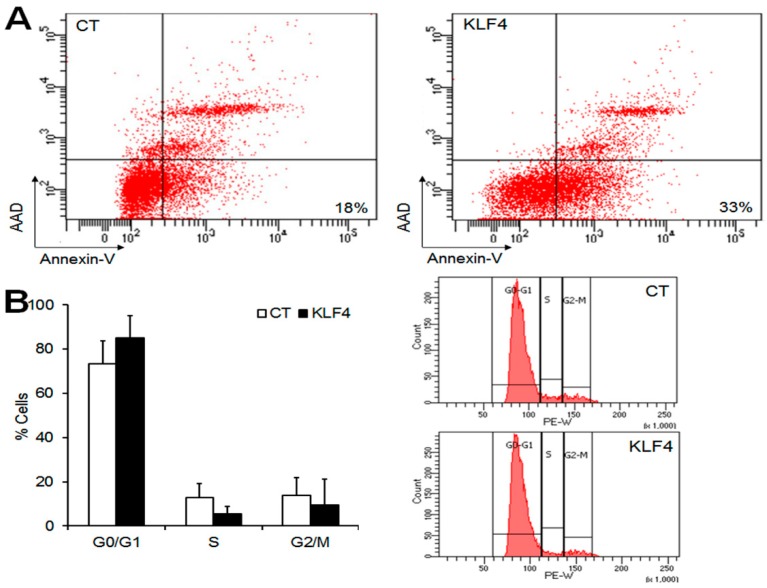
Effect of *Klf4* on apoptosis and the cell cycle of preovulatory GCs in vitro. Flow cytometry assays were performed to analyze apoptosis and the cell cycle in preovulatory GCs overexpressing *Klf4*. (**A**) The flow cytometric detection of apoptosis in GCs transfected with empty vector (CT, control) (left panel), or *Klf4* expression vector (300 ng) (right panel). The cells were stained with Annexin-V and 7-aminoacinomycin D (AAD). The percentages indicate the proportions of late apoptotic cells (right lower quadrant) (Annexin-V+/AAD−). Representative images of three separate experiments are shown; (**B**) Analysis of the subpopulations of cells in cell cycle phases G0/G1, S, and G2/M. The cell cycle distributions were calculated and expressed as means ± SDs of three independent experiments. Representative Fluorescence-activated cell sorting (FACS) profiles are shown (right panel). CT, control cells transfected with empty vector; KLF4, cells transfected with *Klf4* (300 ng).

As *Klf4* overexpression reduced the expression of anti-apoptotic factors and cell growth, we examined its impact on apoptosis and the cell cycle in GCs. To estimate the pro-apoptotic effect of *Klf4* in GCs, apoptotic cells were measured by Annexin V and 7-aminoacinomycin D (AAD) staining and the flow cytometry of GCs transfected with *Klf4* or an empty vector. The percentage of apoptotic cells (Annexin V+/AAD−) increased up to 33% in *Klf4*-transfected cells compared with empty vector-transfected controls (18%) (Figure 4A). As shown in Figure 4B, *Klf4* overexpression also increased the proportion of G0/G1 phase cells (CT, 73.3 ± 10.4 vs. *Klf4*, 84.9 ± 10.1%), and reduced the proportion of S-phase cells compared to the control (CT, 12.7 ± 6.4 vs. *Klf4*, 5.5 ± 3.2%), although statistical significance was not attained (vs. CT, *p* = 0.05). The percentage of cells in the G2/M phase was comparable in the two groups. These results indicate that *Klf4* is involved in the induction of apoptosis, and cell cycle arrest in G0/G1, in preovulatory GCs.

## 3. Discussion

The transient induction of specific transcription factors in periovulatory follicles by the LH surge play an important role in cellular division, apoptosis, and differentiation [3]. In this study, we showed that LH significantly increased *Klf4* expression in preovulatory GCs, and that *Klf4* inhibited cell growth and induced apoptosis in these cells, indicating a role for *Klf4* as a regulator of cell growth and apoptosis during the periovulatory period. 

Using DNA chip technology, the *Klf4* gene has been identified in mice ovarian follicles as one of the folliculogenesis-related genes [13], and its transcript level was markedly increased in Graafian follicles compared to preantral follicles [13]. In addition, LH stimulation rapidly increased *Klf4* transcripts within 2 h in porcine granulosa-lutein cells in vitro [9]. Consistent with this, we observed that LH treatment led to a dose-dependent increase of the *Klf4* transcript level in preovulatory GCs (Figure 1A), suggesting that the ovulatory LH stimulus could be an initial trigger of *Klf4* expression. 

Luteinizing GCs exit the cell cycle and stop dividing, and this is accompanied by the rapid down-regulation of the cell cycle gene, *cyclin D2*, as well as activation of the cell cycle inhibitors, *p21* and *p27* [14]. Our results confirmed that ovulatory doses of LH decreased the expression of *cyclin D2* and increased *p21* mRNA in GCs, with no significant change in *p27* mRNA (Figure 1C). We also observed a decrease in the expression of *cyclin D1*, which is another cell cycle gene [15]. 

Since *Klf4* has been known to cause G1/S arrest through the transcriptional activation of *p21* or *p27* and by the direct inhibition of *cyclin D1* in various types of non-gonadal cells [16,17,18,19,20], we analyzed the expression of these cycle regulators. In addition, *cyclin D2*, a potent activator of the cell cycle in the G1/S phase, was also evaluated because it is selectively expressed in the GCs of growing follicles in the ovary [14]. As expected, *Klf4* overexpression decreased *cyclins D1* and *D2* transcripts and increased those of *p21* in preovulatory GCs (Figure 2B), and *Klf4* knockdown increased *cyclin D2* expression (Figure 2D). However, *Klf4* knockdown had no effects on the *p21* mRNA expression; considering that *Klf4* expression was not completely blocked by siRNA treatment in the present study (Supplementary Figure S1-B,D), it is possible that only the complete absence of *Klf4* would have an effect on *p21* gene expression. 

In agreement with the effects in cell cycle regulators, the overexpression of *Klf4* inhibited GC proliferation (Figure 3D) and cell cycle progression at the G1/S transition (Figure 4B). *p27* is also known as a potent inhibitor of cyclin-dependent kinases in the G1 phase of the cell cycle and its mRNA increases in response to *Klf4* overexpression in non-gonadal cells [16,17,18,19,20,21]. However, changes in *Klf4* expression had no effects on *p27* mRNA levels in GCs (Figure 2B,D). Since *p27* is known to be regulated primarily at the post-translational level [22,23], its mRNA level may not change after *Klf4* induction in GCs.

We also characterized the LH-induced changes in the expression of the *Bcl-2* proto-oncogene (an inhibitor of apoptosis) and *Bax* (an inducer of apoptosis), which are involved in GC apoptosis [24]. The observed LH-induced decrease in *Bcl-2* expression coupled with constant *Bax* mRNA levels (Figure 1B) may be associated with the transient induction of GCs apoptosis before the luteal transition. Since periovulatory GCs are more prone to apoptosis than immature GCs [25,26,27], *Klf4* may also be implicated in the increased susceptibility of preovulatory GCs to apoptosis during this transition period. In fact, evidence from various mammalian cell types indicates that *Klf4* induces apoptosis by regulating the transcription of *Bax* and *Bcl-2* [10], and we also observed that *Klf4* overexpression decreased in *Bcl-2* expression without changing *Bax* mRNA levels, thus lowering the *Bcl-2*/*Bax* ratio (Figure 2A). Another important regulator of the apoptotic process is the caspase family of proteases [28]. The increased permeability of mitochondria under the influence of *Bax* results in the activation of caspases 3 and 7 as the final common pathway of apoptosis [29,30]. In addition to *Bcl-2* and *Bax* regulation by *Klf4* in GCs, we demonstrated the pro-apoptotic effects of *Klf4* as shown in Figure 3C, although these were not statistically significant. Along with this, *Klf4* overexpression reduced cell viability and increased the proportion of apoptotic cells (Figure 3A and Figure 4A). Previous studies also found that *Klf4* overexpression suppressed *Bcl-2* expression in mice small follicles and in the GCs isolated from them [10]. However, they also reported the *Klf4*-induced activation of *Bax* expression. This discrepancy may be due to the differences in cell maturity or sensitivity to apoptosis as a function of follicle growth. Since *Klf4* is highly expressed in Graafian follicles [13] and regulated by LH treatment in GCs, using preovulatory GCs might be more appropriate for analyzing the effect of *Klf4* on GCs than whole ovaries or small follicles. In ovarian cancer cells, *Klf4* overexpression increased *Bcl-2* expression, but had no effect on cell viability [31]. Previous studies indicate that *Klf4* may switch from anti-apoptotic to pro-apoptotic depending on the context [32,33,34]. Thus, *Klf4* seems to regulate *Bcl-2* or *Bax* differently in ovarian cancer cells than in normal ovarian cells due to unidentified cellular factors. 

Considering that the massive apoptosis of preovulatory GCs in vitro is suppressed by an ovulatory dose of LH [35], redundant mechanisms to ensure their survival may exist. Follicle maturation and ovulation are critically dependent on gonadotropins that work in concert with intraovarian factors to suppress the cell death program [36]. The signaling pathway and potencies of various survival factors are different according to the follicle growth [36]. For instance, the activation of the Phosphoinositide 3-kinase/Protein kinase B (PI3K/Akt) pathway by ghrelin is involved in the regulation of cell survival and anti-apoptotic protection in bovine cumulus cells [37,38].

Collectively, our results suggest that *Klf4* may influence the susceptibility of preovulatory GCs to apoptosis at a number of points, including the regulation of upstream regulator (down-regulating *Bcl-2*, thereby decreasing the *Bcl-2*/*Bax* ratio) and downstream effectors (*caspase 3* and *7*). In addition, *Klf4* may contribute to the LH-induced cell cycle exit by altering cell cycle regulators at the G1/S checkpoint after the LH surge, and some time before the complete luteal transition. Thus, *Klf4* is a transcriptional regulator influencing the fate of preovulatory GCs by reducing cell growth and increasing apoptosis during the luteal transition.

## 4. Materials and Methods

### 4.1. Animals and Reagents

Immature female Sprague–Dawley rats were obtained from Orient Biokorea (Kyunggi-do, South Korea) and housed under controlled conditions (22–24 °C, humidity 40–50%, 12-h light–dark cycle), with free access to food and water. Animal care was consistent with institutional guidelines, and the Hanyang University Animal Care and Use committee approved all of the procedures involving animals (HY-IACUC-16-0013, approval date: 24 February 2014). McCoy’s 5a medium (modified) and Leibovitz L-15 medium were obtained from GIBCO (Santa Clara, CA, USA). Penicillin and streptomycin were obtained from Sigma (St. Louis, MO, USA) and recombinant human FSH (Org 32489E) and ovine LH (NIH-LH-23) were from NV Organon (Oss, The Netherlands) and the National Hormone and Pituitary Distribution Program (Baltimore, MD, USA), respectively.

### 4.2. Preparation and Culture of Preovulatory Granulosa Cells

Animals aged 26 days (body weight 55–60 g) were injected intraperitoneally with 10 International Unit (IU) of pregnant mare serum gonadotropin (PMSG) (Sigma-Aldrich, St. Louis, MO, USA) to induce the growth of multiple preovulatory follicles. Forty-eight h later, ovaries were dissected, and preovulatory follicles were punctured to obtain granulosa cells. Ovarian debris and small follicles were removed, and GCs were collected by low-speed centrifugation at 500 × *g* for 10 min, dispersed by repeated washing and suspended in culture medium (McCoy’s 5a supplemented with two mM of L-glutamine, 100 U/mL of penicillin, and 100 μg/mL of streptomycin). To evaluate the effect of LH on expression of *Klf4* on apoptosis-related and cell cycle-related genes, cells were plated in a 24-well culture dish (5 × 10^4^ viable cells/well) and cultured in the absence or presence of LH (100 and 200 ng/mL) for 45 min. At the end of the experiment, they were frozen for RNA extraction. 

### 4.3. Plasmid Construction and Transfection

The pCMV3×FLAG-*Klf4* (FLAG-*Klf4*) construct was made by subcloning full-length rat *Klf4* cDNA (kindly provided by H. Kook; Chonnam National University Medical School, Gwangju, Korea). *Klf4*-specific siRNA (*Klf4*-siRNA) was synthesized by Genepharma (Shanghai, China); the sequences of the 25-nucleotide sense and antisense RNAs were: 5′-CCAUUAUCAAGAGCUCAUGCCACCG-3′ (sense) and 5′-CGGUGGCAUGAGCUCUUGAUAAUGG-3′ (antisense) (accession no.: NM_053713). Non-targeting control siRNA (Universal Scrambled siRNA) was purchased from Genepharma (Shanghai, China). For transient transfections, GCs were resuspended in electroporation buffer (MPK1025; Thermo Fisher Scientific, Waltham, MA, USA), mixed with expression vectors or siRNA, and electroporated with a single pulse of 1000 V, 40 milliseconds, using a Neon™ Transfection System (MPK5000, Thermo Fisher Scientific, Waltham, MA, USA). This procedure was based on the results of preliminary optimization experiments that achieved a transfection efficiency of approximately 70%. The transfected cells (1 × 10^5^ cells/well) were resuspended in culture medium, plated in 24-well culture plates, collected after 24–36 h, and snap-frozen for the isolation of RNA or proteins. 

### 4.4. Total RNA Extraction and Real-Time Quantitative RT-PCR Analysis

Total RNA was isolated with an RNeasy extraction kit (Qiagen Inc., Valencia, CA, USA). One-µg aliquots of total RNA were annealed (five min at 70 °C) to oligo(dT)_18_ primers and reverse transcribed using cDNA synthesis platinum master mix (GenDEPOT, Katy, TX, USA). Primers were designed for the mRNA sequences of *Klf4*, *Bax*, *Bcl-2*, *cyclin D1*, *cyclin D2*, *p21*, and *p27* using the Primers Express program (PE Applied Biosystems, Foster City, CA, USA) (Table 1). Real-time PCR reactions were carried out in total volumes of 20 μL, with Prime Q-Master Mix (with SYBR Green I) (GeNet Bio Inc., Daejeon, South Korea) using a LightCycler 480 II System (Roche Molecular Diagnostics, Indianapolis, IN, USA). The PCR cycle conditions were as follows: 10 min at 95 °C, 45 cycles of 95 °C for 10 s, 58~60 °C for 10 s, and 72 °C for 10 s. *18S rRNA* was amplified and used to normalize each reaction. Samples were run in triplicate (Roche Molecular Diagnostics, Indianapolis, IN, USA), and mean values were compared with the control values to calculate relative amounts of transcript. Data are expressed as means ± standard deviations (SDs) of duplicate or triplicate measurements in four independent experiments. 

### 4.5. Western Blot Analysis

Transfected GCs were lysed in Laemmli buffer containing β-mercaptoethanol (BioRad, Hercules, CA, USA). Samples were resolved by 8% Sodium dodecyl sulfate-polyacrylamide (SDS-PAGE) gel electrophoresis, transferred to nitrocellulose membranes (Amersham Pharmacia Biotech, Arlington Heights, IL, USA), and immunoblotted with anti-KLF4 antibody (4038, Cell Signaling Tech., Inc., Danvers, MA, USA) diluted 1:200. The membranes were washed and blotted with peroxidase-conjugated donkey anti-rabbit secondary antibody (1:2000) (Boehringer Mannheim, Indianapolis, IN, USA). Immunolabeled proteins were detected using an enhanced chemiluminescence kit (Pierce Chemical Co., Rockford, IL, USA). The 65-kDa KLF4 protein is indicated in Supplementary Figure S1D. To ensure that the lysates were loaded equally, the blots were stripped and incubated with an anti-β-actin antibody (1:4000) (Sigma, St. Louis, MO, USA).

### 4.6. Cell Counting Kit-8 Assays

Cell viability was determined using a CCK-8 (Enzo, Ann Arbor, MI, USA). Dehydrogenase enzymes present in metabolically active cells convert water-soluble tetrazolium-8 salt (WST-8) [2-(2-methoxy-4-nitrophenyl)-3-(4-nitrophenyl)-5-(2,4-disulfophenyl)-2H-tetrazolium, monosodium salt] to a water-soluble formazan dye. The amount of the formazan dye that is generated is proportional to the number of living cells. GCs transfected with empty vector (EV) or Flag-*Klf4* (300 ng/well) were plated in 96-well plates (1 × 10^4^ cells/well) and cultured in McCoy 5a medium containing 1% fetal bovine serum (FBS). Sixteen to 24 h after transfection, WST-8 solution was added and incubation continued in serum-free medium for 2 h. The quantity of formazan product was measured at 450 nm with an ELISA plate reader (BioRad, Hercules, CA, USA). The experiment was repeated three times, and the data are expressed as fold changes relative to the control values measured with empty vector-transfected cells. Cell viability was evaluated using trypan blue staining [39]. Equal volumes of 0.4% trypan blue were added to cell suspensions, and viable (unstained) and dead (stained) cells were counted using a hemocytometer. The data are expressed as percentages of viable cells. All of the experiments were replicated at least three times to assure reproducibility (Louis, MO, USA).

### 4.7. Caspase 3/7 Assays

Apoptosis was also assessed with the caspase 3/7 activity assay using a luminescence assay kit (Caspase-Glo 3/7 assay; Promega, Madison, WI, USA). GCs were cultured in McCoy 5a serum-free medium with or without FSH or 10% FBS for 24 h after transfection. Then, proluminescent Caspase-Glo 3/7 reagent was added to each well, and the cells were gently mixed and incubated at room temperature for one h. Luminescence was measured on a Monolight 2010 luminometer (Analytical Luminescence Laboratory, San Diego, CA, USA). The data were expressed as fold changes relative to the empty vector transfected cell (control). All of the experiments were replicated three times. 

### 4.8. 5-Bromo-2′-Deoxyuridine (BrdU) Incorporation

Cells were plated onto 1.2 × 1.2 cm coverslips in 24-well plates (1 × 10^5^ cells/well). After 16–24 h, 5-bromo-2′-deoxyuridine (BrdU, 3 µg/mL) (B5002, Sigma, St. Louis, MO, USA) was added and incubation continued for three h, followed by fixation with methanol for 10 min at −20 °C and three washes with phosphate-buffered saline (PBS). The slides were dehydrated, pretreated with 2N HCl at 37 °C for 15 min, washed twice with 0.2% Triton X in PBS (pH 7.4), and incubated with 1% normal goat serum/PBS for one h at room temperature, followed by anti-BrdU antibody (B2531, Sigma, St. Louis, MO, USA) diluted 1:250 in 1% normal goat serum at 4 °C overnight. The slides were then washed with PBS and incubated with 1:200 goat anti-mouse Alexa Fluor 488^®^ IgG (Thermo Fisher Scientific, Waltham, MA, USA) for one h at 37 °C, and nuclear DNA was counterstained with 4′,6-diamidino-2-phenylindole (DAPI) (Vector Laboratories, Inc., Burlingame, CA, USA). Five magnified fields (100×) were randomly selected, and the number of BrdU-positive cells per field was counted under a fluorescence microscopy (Leica, Heidelberg, Germany). The proliferation rate (%) was defined as the percentage of total cells that were BrdU-positive. 

### 4.9. Flow Cytometric Analysis

For assessing apoptosis, cells were washed twice with cold PBS, resuspended in 1X binding buffer (1 × 10^6^ cells) (BD Biosciences, San Jose, CA, USA) and stained with APC-Annexin V and 7-AAD (BD Biosciences, San Jose, CA, USA) for 15 min in the dark at room temperature. Unstained cells were used to set negative gates, and each cell that was stained with either Allophycocyanin (APC)-Annexin V or 7-Aminoactinomycin D (7-AAD) alone was used to set the compensation parameters. Apoptosis rates were measured using a flow cytometer (FACSCalibur, BD Biosciences, San Jose, CA, USA) and analyzed with Cell Quest software (BD Biosciences, San Jose, CA, USA). 

For cell cycle analysis, cells (1 × 10^6^ cells) were harvested, fixed in 70% (*v*/*v*) ethanol for two h at −20 °C, washed twice with PBS, treated with RNase A (iNtRON Biotechnology, Seoul, Korea), and stained with propidium iodide (Sigma, St. Louis, MO, USA) at room temperature for 30 min in the dark. Using flow cytometry, the proportion of cells in each phases (G0-G1, S, and G-M) were analyzed. 

### 4.10. Statistical Analysis

All of the results were expressed as means ± SDs of experiments and repeated at least three times. All of the data were analyzed using IBM SPSS statistics 21 for Windows (SPSS Inc., Chicago, IL, USA). Statistical significance was determined by the Mann–Whitney U test for two-group comparisons, and Kruskal–Wallis one-way analysis of variance for multiple comparisons. Significance was accepted at *p* < 0.05. 

## 5. Conclusions

We conclude that *Klf4* increases the susceptibility of preovulatory GCs to apoptosis by down-regulating *Bcl-2* and promotes LH-induced cell cycle exit. It appears to be a key regulator induced by the LH surge that determines the fate of GCs in preovulatory follicles during the luteal transition.

## Figures and Tables

**Table 1:** Primer sequences used for real-time PCR amplification.

Gene	Primer Sequence (5′-3′)	Product Size (bp)	Accession Number
*Klf4*	F: GAGAGGAACTCTCTCACATGAAGC	185	NM_053713.1
	R: AAGGATAAAGTCTAGGTCCAGGAGA		
*Bax*	F: TGTTTGCTGATGGCAACTTC	104	NM_017059.1
	R: GATCAGCTCGGGCACTTTAG		
*Bcl-2*	F: GGGATGCCTTTGTGGAACTA	138	NM_016993.1
	R: CTCACTTGTGGCCCAGGTAT		
*Cyclin D1*	F: TCAAGTGTGACCCGGACTG	213	NM_171992.4
	R: CACTACTTGGTGACTCCCGC		
*Cyclin D2*	F: CGATGATCGCAACTGGAAGC	232	NM_022267.1
	R: TGGTCCGGATCTTCCACAGA		
*p21^Cip1^*	F: TGAGGGACCAGTACATGAGAACT	192	NM_031515.1
	R: GAGCCTGTTTCGTGTCTACTGTT		
*p27^Kip1^*	F: TCGCGGCTCCGAGACTT	204	NM_031762.3
	R: TCTCCAAGTCCCGGGTTAGT		
*18S rRNA*	F: CGCGGTTCTATTTTGTTGGT	218	M11188.1
	R: AGTCGGCATCGTTTATGGTC		

F, forward; R, reverse; *p21^Cip1^*, cyclin-dependent kinase-interaction protein 1; *p27^Kip1^*, cyclin-dependent kinase inhibitor 1B.

## Supplementary Materials

Supplementary materials can be found at http://www.mdpi.com/1422-0067/20/1/87/s1.
