# Supplementary material for: Role of Klf4 in the Regulation of Apoptosis and Cell Cycle in Rat Granulosa Cells during the Periovulatory Period
Source: Int J Mol Sci. 2018 Dec 26;20(1):87. doi: 10.3390/ijms20010087 (PMC6337711; doi:10.3390/ijms20010087)
Supplement: Supplementary file 1 [file ijms-20-00087-s001.pdf]

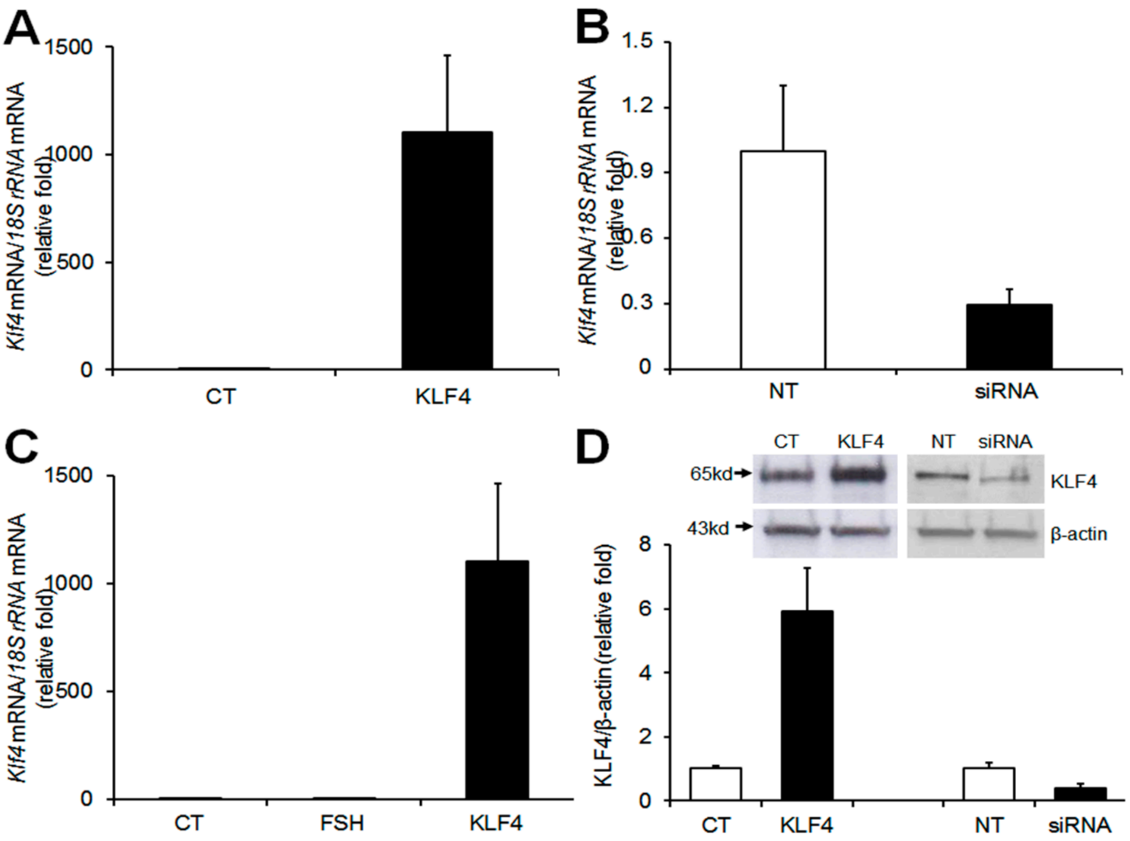

Supplementary Figure S1. Real-time PCR and immunoblot analysis of *Klf4* mRNA and protein in GCs to confirm (**A**,**C**,**D**) *Klf4* overexpression and (**B**,**D**) *Klf4* knockdown. *Klf4* mRNA levels were normalized with *18S rRNA* levels. Bars indicate fold changes relative to control, and values are means ± SDs of data pooled from three independent experiments. (**D**) In the immunoblot analysis, bands corresponding to *Klf4* (65 kDa) and *β-actin* (43 kDa) were indicated, respectively (upper panel). The density was transformed to pixels, and the digitized images were analyzed using Gel Plot 2 Macro for Scion Image beta 4.0.2 software (Scion Corporation, Frederick, MD, USA). Each bar indicates the fold change to the value for CT or NT, and represents the mean ± SD from two independent experiments (lower panel). CT, control cells transfected with empty vector; KLF4, cells transfected with *Klf4* (300 ng); NT, cells transfected with non-target siRNA; siRNA, cells transfected with *Klf4*-specific siRNA (200 nM).
